# 

**Published:** 1807-05

**Authors:** 


### NEW MEDICAL PUBLICATIONS.

A Popular Essay, on the Disorder familiarly termed a Cold; by E. L. White. 8vo. 5s boards

Strictures on Mr. Parkinson's Observations, on the Nature and Cure of the Gout, recently published in Opposition to the Theory that proposes the Cooling Treatment of that Disease; to which are added in an Appendix, Two Letters addressed to Dr. Haygarth; by Robert Kinglake, M. D. 4s. boards.

Dr. Trotter's View of the Nervous Temperament; being a practical Enquiry into the increasing Prevalence, Prevention and Treatment of those Diseases, commonly called Nervous, Bilious, Stomach, and Liver Complaints, Indigestion, Low Spirits, Colic, Gout, &c.

Address to the Professors of Physic and Surgery in London and Westminster, proposing the Institution of a Society for investigating the Cause, Symptoms and Cure of the Hydrophobia. 6d.

Observations on the Application of Lunar Caustic to Strictures of the Urethra and Œsophagus, by M. W. Andrews, M. D. Member of the Royal College of Surgeons in London, late Army Surgeon, and now Physician at Madeira, 5s 6d.

---

### To CORRESPONDENTS.

A general Abstract of Persons Vaccinated at the Presidency of Madras, from Sept. 1805 to Aug. 1806, amounting to 178,074, will be inserted in our next Number.

Dr. Bellamy's Essay on Military Hospitals shall be sent as desired.

Communications are received from Dr. M'Grigor, &c.

